# Advances in Materials for Strontium–Yttrium Separation: A Comprehensive Review

**DOI:** 10.3390/ma19132887

**Published:** 2026-07-06

**Authors:** Mali Xu, Zhimin Wang, Tong Zhang, Siqi Ma, Shengyang Zhao, Yonggang Zhao, Yan Chen

**Affiliations:** Department of Radiochemistry and Engineering, China Institute of Atomic Energy, P.O. Box 275-88, Beijing 102413, China; maryxu2013@163.com (M.X.); zhimin8396@163.com (Z.W.); zhangtonga@sohu.com (T.Z.); msq1994pe@126.com (S.M.); zhaosy@cnncmail.cn (S.Z.)

**Keywords:** ^90^Sr-^90^Y generators, adsorbent materials, extraction chromatography, ion exchange resins, medical isotopes

## Abstract

Yttrium-90 (^90^Y) is a pivotal pure beta-emitting radionuclide extensively employed in the targeted therapy of malignant tumors, such as hepatocellular carcinoma and lymphoma. The ^90^Sr-^90^Y generator system represents the most effective method for producing no-carrier-added (NCA) ^90^Y to meet escalating clinical demands. However, safe clinical application necessitates the stringent separation of its parent isotope, ^90^Sr, which poses significant radiotoxicological risks due to its long half-life and bone-seeking behavior. This review comprehensively summarizes recent advances in solid-phase adsorbent materials developed for the high-efficiency separation of Y^3+^ and Sr^2+^. We systematically analyze the design strategies, molecular recognition mechanisms, and performance evaluation metrics of various functional systems. Key materials discussed include extraction chromatography (EXC) resins based on organophosphorus extractants, diglycolamide (DGA) derivatives, and crown ethers, as well as inorganic ion exchangers such as antimony-based materials, manganese oxides, and zeolite-like molecular sieves. Special attention is given to composite modification strategies, including silica-based and polymer-matrix composites, and metal doping techniques aimed at enhancing radiation resistance, acid stability, and Sr-Y separation factors (SF). Finally, we provide an outlook on the future development of next-generation ^90^Sr-^90^Y generator materials, highlighting the imperative of transitioning from idealized simulated environments to robust, field-ready applications.

## 1. Introduction

^90^Y is a pure beta emitting radionuclide characterized by a half-life of 64.1 h and a maximum beta energy of 2.28 MeV, decaying to stable ^90^Zr without gamma emission [1,2]. With a mean tissue penetration of 2.5 mm and a range of 11 mm [3], ^90^Y delivers a potent therapeutic dose that effectively eradicates tumor cells while sparing surrounding healthy tissue. It has become an important therapeutic nuclide in targeted therapy, widely used in the treatment of various malignant tumors such as hepatocellular carcinoma and lymphoma [2,4]. The escalating global clinical demand for ^90^Y necessitates robust and scalable supply methodologies. Current production pathways primarily involve two distinct technical routes. The first is reactor irradiation, which utilizes the stable isotope ^89^Y as a target material to directly produce ^90^Y via the ^89^Y (n, γ)^90^Y nuclear reaction, a method yielding relatively low specific activity that poses challenges for direct radiolabeling [5]. The second is radionuclide generator, which uses parent ^90^Sr (T_1/2_ = 28.8 y) from uranium fission products to continuously generate ^90^Y through β^−^ decay, and then obtains NCA ^90^Y from ^90^Sr through separation and purification techniques [6,7,8,9,10,11]. The latter is considered more attractive due to the superior specific activity and dosimetric precision of the produced NCA ^90^Y [2,11]. However, the major challenge with ^90^Sr-^90^Y generators is ensuring the radiochemical purity of ^90^Y, as ^90^Sr is a bone-seeker with a long half-life, and its presence can lead to prolonged bone marrow irradiation and myelosuppression in patients [12]. The International Commission on Radiological Protection (ICRP) recommends that the ^90^Sr content in ^90^Y preparations for human use should be less than 2 × 10^−3^% of the total activity [13]. The production of medical-grade ^90^Y imposes stringent requirements on ^90^Sr removal, posing a significant challenge to the efficiency and selectivity of the separation process.

Traditional separation techniques, such as liquid–liquid extraction [7,14,15,16], precipitation [6,17], and electrode position [18,19,20], often limited both in speed and selectivity, rendering them suboptimal for rapid clinical production. Consequently, solid-phase adsorbent materials have emerged as the preferred option for ^90^Sr-^90^Y separation owing to their operational simplicity, high column efficiency, and excellent chemical and radiolytic stability [21,22,23]. A variety of solid adsorbents have been developed, including extraction chromatography resins, organic ion-exchange materials, and inorganic ion exchangers, demonstrating considerable potential for generator applications.

Several review articles have summarized advances in ^90^Sr-^90^Y generator technologies and radionuclide separation methods. However, most existing reviews focus on the overall generator systems, encompassing a broad range of separation approaches such as electrochemical methods, precipitation, solvent extraction, and chromatographic techniques [1,2,24]. Other reviews primarily address radionuclide separation and purification in the context of the nuclear fuel cycle [25,26]. In contrast, comprehensive reviews specifically dedicated to solid-phase adsorbent materials for ^90^Sr-^90^Y generators remain scarce. Furthermore, comparisons among different classes of adsorbent materials are often qualitative and fragmented, making it difficult to identify the key factors governing separation performance and practical applicability.

In addition, most reported studies have been conducted under idealized laboratory conditions using stable isotopes and batch adsorption experiments. Limited attention has been paid to the separation of trace amounts of ^90^Y from high-activity ^90^Sr matrices, which represents the actual operating condition of radionuclide generators. The relationship between fundamental material properties, such as distribution coefficient (K_d_), SF, and adsorption capacity, and clinically relevant requirements for ^90^Y production has also not been systematically established.

Therefore, this review focuses specifically on solid-phase adsorbent materials employed in ^90^Sr-^90^Y generators. The review provides a systematic comparison of extraction chromatography resins, organic ion exchangers, and inorganic ion exchangers, analyzes the structure–performance relationships governing Sr/Y separation, and discusses their advantages and limitations under generator-relevant conditions. Particular emphasis is placed on linking material performance to the stringent radiochemical purity requirements for medical-grade ^90^Y and identifying the remaining challenges that must be addressed for future clinical applications.

**Figure 1:**
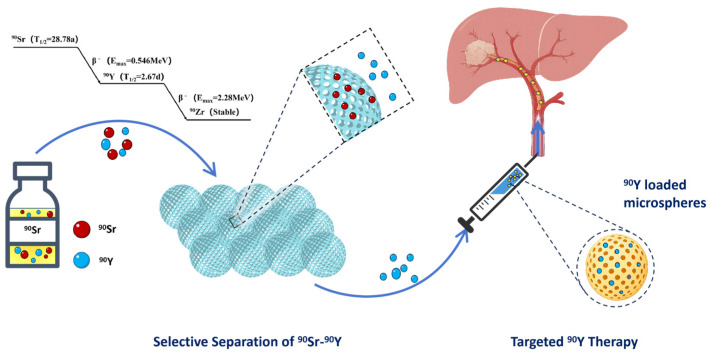
Conceptual schematic of solid adsorbent-based ^90^Sr-^90^Y separation for medical ^90^Y production.

Figure 1 schematically illustrates the selective separation of ^90^Sr-^90^Y using solid adsorbent materials and the subsequent application of purified ^90^Y in targeted radionuclide therapy.

## 2. Extraction Chromatography Resins

EXC combines solvent extraction and chromatographic separation by immobilizing highly selective extractants, typically derived from liquid–liquid extraction systems, onto inert porous supports [27,28,29]. By retaining the high selectivity of solvent extraction while offering the operational simplicity, high column efficiency, and ease of automation characteristic of chromatography, this technique has emerged as a pivotal approach for preparing high-purity ^90^Y in ^90^Sr-^90^Y generators. The separation performance of extraction chromatographic resins is primarily determined by the selectivity and extraction behavior of the incorporated extractants. The following sections provide an in-depth overview of recent advances in ^90^Sr-^90^Y separation based on different classes of extractants and adsorption materials.

### 2.1. EXC Resins Based on Organo-Phosphorous Extractants

**Figure 2:**
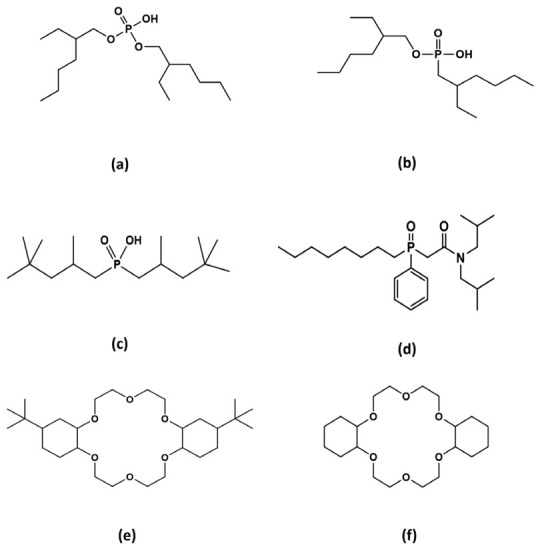
Structural formula of the extractant: (**a**) HDEHP; (**b**) HEH[EHP]; (**c**) H[DTMPP]; (**d**) Octyl(phenyl)-N,N-diisobutylcarbamoylmethylphosphine oxide (CMPO); (**e**) Di-tert-butylcyclohexano-18-crown-6 (DtBuCH18C6); (**f**) Dicyclohexano-18-crown-6 (DCH18C6).

Acidic organophosphorus extractants play a pivotal role in the ^90^Sr-^90^Y separation system, particularly in the context of extraction chromatography. Their molecular recognition mechanism is primarily driven by cation exchange and shows high sensitivity to changes in pH. This allows for the efficient separation of Sr^2+^ and Y^3+^ via accurate adjustment of conditions. Figure 2a–c depicts typical extractants in this category, such as di(2-ethylhexyl) phosphoric acid (HDEHP), 2-ethylhexyl phosphonic acid mono-2-ethylhexyl ester (HEH[EHP]), and bis(2,4,4-trimethylpentyl) phosphinic acid (H[DTMPP]) [30]. Due to their superior affinity for Y^3+^ compared to Sr^2+^, these extractants are widely utilized in Sr-Y separation processes. Notably, E.P. Horwitz and his team developed the commercial LN, LN2, and LN3 resin series by impregnating these specific extractants onto the Amberchrom CG-71 acrylic polymer matrix [31,32]. 

HDEHP is one of the most widely utilized extractants in the separation and recovery of lanthanides and other metal ions through solvent extraction and extraction chromatography [27]. HDEHP-based EXC resins have been specifically studied for the separation of ^90^Y from ^90^Sr [7,21,33]. HDEHP achieves selective extraction via a cation exchange mechanism, in which the acidic P-OH group coordinates with metal cations while the P=O group facilitates the extraction process [34]. Studies by Peppard et al. [35] demonstrated that acidic dialkyl organophosphorus extractants form highly stable dimers in hydrocarbon diluents via hydrogen bonding. The typical ion exchange reaction occurring between rare earth metals and HDEHP is described as follows:

(1) $$M^{3+}+3{\left(HA\right)}_{2}\Leftrightarrow [M{\left(X\right)}_{3}{\left(HX\right)}_{3}]+3H^{+},$$

where M stands for a trivalent rare earth metal ion, and HA represents HDEHP.

Extensive studies have demonstrated that HDEHP exhibits a significantly higher distribution ratio (D) for Y^3+^ than for Sr^2+^ [31]. Early work by Hsieh et al. [7] utilized HDEHP immobilized on polytetrafluoroethylene (PTFE) particles. Their system employed a dual-column in HCl media to isolate high-purity ^90^Y. Similarly, Shapovalov [36] developed an extraction-chromatographic separation of ^90^Sr and ^90^Y using columns packed with resins impregnated with HDEHP. To achieve medical-grade purity, the protocol incorporated a secondary purification step using an yttrium specific adsorption resin, which consists of CMPO as the active component supported on a TBP matrix, and a cation exchange resin, yielding 64–72% recovery with ^90^Sr breakthrough levels below 10^−8^.

Kim and Ito [37,38] advanced this approach by preparing silica-based resins, specifically (HDEHP + Dodec)/SiO_2_-P and (HDEHP + Oct)/SiO_2_-P, using 1-dodecanol and 1-octanol as molecular modifiers. Their comparative studies in HCl and HNO_3_ media revealed that Y^3+^ adsorption follows a Langmuir monolayer model and is more efficient in HNO_3_. While Taiga Kawamura et al. [39] reported comparable adsorption behavior using 1-hexanol as a modifier. Although these studies provided valuable insights into the adsorption mechanisms and separation performance of HDEHP based materials, their evaluations were performed exclusively under old conditions using stable isotopes, without validation in actual ^90^Sr-^90^Y systems.

Emam et al. prepared a Cyanex 572 impregnated Amberlite XAD-4 resin (Cy–572@XAD–4) for the separation of Sr^2+^, Y^3+^, and Zr^4+^ in hydrochloric acid media [40]. At 0.05 M HCl, the resin exhibited excellent selectivity toward Zr^4+^, with separation factors of 16.4 for Zr/Y and 30.5 for Zr/Sr, and an adsorption affinity order of Zr^4+^ > Y^3+^ > Sr^2+^. The maximum adsorption capacities fitted by the Langmuir model were 29.1, 9.3, and 6.1 mg·g^−1^, respectively. Column experiments with stepwise elution achieved the separation of the three elements. Sr^2+^ was removed with water, Zr^4+^ was eluted with 0.5 M oxalic acid, and Y^3+^ was recovered using a mixture of 3.0 M HCl and 0.25 M NaCl, with recovery rates approaching 99% for all three elements, demonstrating efficient one-step separation. This method offers a simple and highly selective chromatographic strategy for the purification of radioactive nuclides such as ^90^Sr, ^90^Y, and ^95^Zr.

CMPO is a neutral, bidentate organophosphorus extractant characterized by the presence of both phosphoryl (P=O) and amide (C=O) functional groups within its molecular structure, as shown in Figure 2c. Common supports include macroporous silica/polymer composites (SiO_2_-P) [41,42], polystyrene-divinylbenzene copolymers (PS-DVB) [42], and PTFE membranes [43]. Among these, SiO_2_-P has emerged as a research focus in recent years due to its combination of the mechanical stability of an inorganic skeleton and the high loading capacity of an organic phase. A typical preparation method involves dissolving CMPO in a diluent, often with a phase modifier such as 1-dodecanol to suppress third-phase formation and improve mass transfer [41]. For instance, Kawamura et al. prepared both CMPO/SiO_2_-P and (CMPO + Dodec)/SiO_2_-P adsorbents. The latter exhibited significantly enhanced adsorption capacity and selectivity for Y^3+^ owing to the addition of 1-dodecanol [41]. Kawamura and colleagues utilized a CMPO/SiO_2_-P packed column, demonstrating that under 4 M HNO_3_ loading conditions, Sr^2+^ exhibits negligible retention while Y^3+^ is effectively retained. Subsequent elution of Y^3+^ using dilute HNO_3_ or complexing agents such as DTPA resulted in recovery yields exceeding 90% [41]. Beyond column chromatography, membrane-based techniques have also been explored. Dhami et al. developed a dual-stage supported liquid membrane (SLM) system. In this setup, the first stage, impregnated with HEH[EHP], was used for the initial extraction of ^90^Y from a ^90^Sr source, while the second stage, containing CMPO, further purified the isotope and transferred it to the receiving phase [44]. This system facilitates the continuous production of NCA ^90^Y and is amenable to automation. In another study, Pareek et al. applied a CMPO/n-dodecane solution within a hollow fiber membrane contactor (HFMC) to ultra-purify ^90^Sr feed solutions by removing α-impurities such as Pu and Am, thereby providing high-purity feedstock for high-quality ^90^Sr-^90^Y generators [45]. Although this study focused on Sr purification, it underscores the versatility of CMPO in membrane platforms.

A critical comparison of the three CMPO-based platforms reveals distinct trade-offs. The SiO_2_-P packed-column configuration offers the simplest operation and has been successfully validated for ^90^Y recovery exceeding 90% [41], making it the most mature option for conventional chromatographic generator systems. The dual-stage SLM system enables continuous production of NCA ^90^Y and is readily adaptable to automated operation [44]. However, the long-term stability of the liquid membrane remains a concern, as gradual loss of the organic phase may reduce transport efficiency during repeated operation. The HFMC approach provides a large interfacial area and enhanced mass-transfer performance [45]; nevertheless, its application has thus far been limited to purification of ^90^Sr feed solutions rather than direct ^90^Y recovery, and its relatively complex engineering requirements may hinder routine implementation.

Despite their different configurations, all three platforms rely predominantly on physically impregnated CMPO and therefore share a common challenge associated with extractant retention. In chromatographic columns, gradual extractant leaching primarily reduces adsorption capacity and may necessitate periodic resin replacement [46,47]. In contrast, extractant loss in membrane-based systems can directly affect transport performance and process stability, making long-term operation more challenging [48]. Although covalent immobilization strategies have been investigated to mitigate extractant loss [49], no practical implementation has yet been reported for Sr-Y separation systems [50]. Consequently, physical impregnation remains the dominant immobilization strategy in current practice, while improving extractant retention continues to be a key challenge for future CMPO-based separation technologies.

Collectively, these studies demonstrate that extraction chromatography materials based on HDEHP, Cyanex 572, and CMPO possess excellent separation performance and considerable potential for application in ^90^Sr-^90^Y generator systems. However, most reported investigations have been conducted under cold conditions using stable isotopes or simulated solutions. As a result, the effects of radiation on material performance remain insufficiently understood. High-energy beta particles emitted during the decay of ^90^Sr and ^90^Y can induce radiolytic degradation of organic extractants, leading to changes in coordination behavior, adsorption capacity, and separation selectivity. Radiation-induced degradation has been reported for several classes of extractants, including organophosphorus compounds, diglycolamides, and crown ethers, indicating that material performance observed in laboratory-scale experiments may not accurately reflect behavior under practical operating conditions [51,52,53,54]. In addition, prolonged operation of high-activity generator systems may result in localized heat accumulation, which can influence mass transfer processes and adsorption equilibria [55,56]. More importantly, stable-isotope studies cannot directly verify whether the radionuclidic purity of the obtained ^90^Y satisfies clinical requirements, particularly the ICRP-recommended limit for ^90^Sr breakthrough of less than 2 × 10^−3^% of the total activity [13]. Therefore, although these materials exhibit promising separation characteristics, systematic validation under realistic radioactive conditions remains essential before their long-term applicability in medical ^90^Sr-^90^Y generators can be fully established.

### 2.2. EXC Resins Containing Crown Ethers

Crown ethers, particularly macrocyclic polyethers like 18-crown-6, possess unique cavity dimensions that enable highly selective complexation with metal ions of specific sizes [57]. For Sr^2+^ with an ionic radius of approximately 1.26 Å, the 18-crown-6 macrocycle offers a complementary fit, forming a stable 1:1 complex that effectively weakens Sr^2+^ solvation and facilitates its extraction [58]. Notably, derivatives such as DtBuCH18C6 and DCH18C6, whose structures are depicted in Figure 2e,f, are distinguished by their ability to isolate Sr^2+^ from highly acidic media while exhibiting negligible affinity for trivalent ions like Y^3+^ [59,60,61]. This molecular recognition mechanism is particularly effective in nitric acid media, as nitrate anions act as counter-ions to stabilize the complex structure, while high nitric acid concentrations help suppress the competitive adsorption of coexisting cations such as Ca^2+^ and Mg^2+^.

This exceptional theoretical selectivity has been successfully translated into practical applications using extraction chromatography. For instance, the resin impregnated with DtBuCH18C6 on a SiO_2_-P support demonstrates this selectivity quantitatively. It exhibits a K_d_ for Sr^2+^ exceeding 182 cm^3^·g^−1^ in 3 M HNO_3_, whereas the value for Y^3+^ remains below 10 cm^3^·g^−1^ [62]. Zou Yu et al. developed an extraction chromatographic resin based on crown ether-ionic liquids, which significantly enhanced Sr^2+^ retention while facilitating the elution of Y^3+^, thereby achieving high-purity preparation of ^90^Y [63]. Similarly, in simulated high-level liquid waste (HLLW), the (DtBuCH18C6 + TBP)/SiO_2_-P composite allows Y^3+^ to elute with the nitric acid wash due to negligible retention, while Sr^2+^ is efficiently retained [64]. For the rapid detection of ^90^Sr in environmental and food samples, Chen et al. developed a fast analytical method utilizing commercial Sr resin [65].

**Figure 3:**
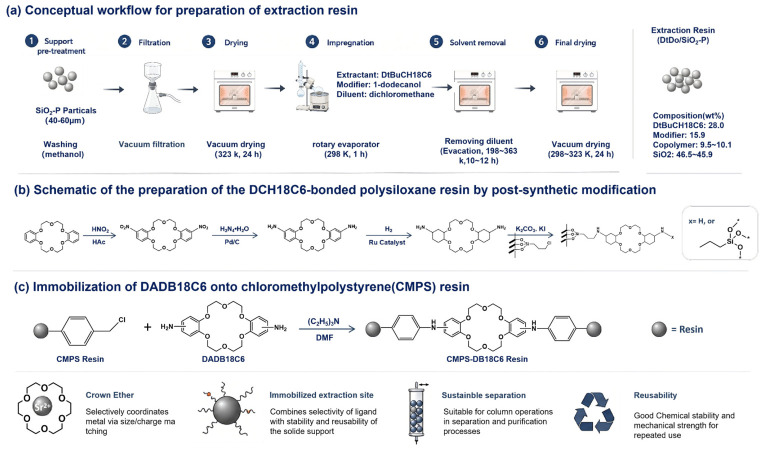
Generalized preparation and immobilization workflow of crown ether-functionalized extraction resins summarized from representative studies. Refs. [67,71,72].

Depending on the binding mode between the crown ether and the support, existing materials are mainly divided into two categories of physical impregnation and chemical bonding. Representative workflows and immobilization routes are summarized in Figure 3. The physical impregnation method involves dissolving the crown ether extractant in an inert diluent and then loading it onto a porous support, which represents the most mature commercial technology route currently available. The Sr resin developed by Horwitz et al., utilizing DtBuCH18C6 as the extractant on Amberlite XAD-7 or Amberchrom CG-71 supports, represents a milestone in this field. This material exhibits an extremely high D_Sr_ in 3 M HNO_3_ media and can effectively separate matrix interferences such as Ca^2+^ and Ba^2+^ [29,66]. Research indicates that the choice of diluent is crucial. Introducing long-chain alcohols [67] (Figure 3a) or TBP [68] not only serves as a solvent but also interacts with the crown ether via hydrogen bonds, thereby suppressing the loss of the crown ether in strong acidic aqueous phases. Furthermore, the solvent-free resin concept proposed by Dietz et al., which involves directly dispersing DtBuCH18C6 onto XAD-7, demonstrates superior physical stability compared to traditional solvent-containing systems [69]. To thoroughly address the issue of extractant leaching, chemically bonded materials covalently graft crown ethers onto support matrices to construct stable solid-phase extraction systems. However, although the bonding was robust, the mass transfer kinetics were sluggish due to the hydrophobicity of the polystyrene. In contrast, Qin and Ye et al. [70,71] employed a solgel method to co-condense crown ether precursors into a silica-based framework, yielding materials with high specific surface area and thermal stability (Figure 3b). A recent study by A.K. Singha Deb et al. [72] has further advanced this field. They synthesized a dibenzo-18-crown-6 functionalized chloromethylated polystyrene (CMPS-DB18C6) resin via a polycondensation reaction (Figure 3c). This material not only effectively overcomes the kinetic limitations of traditional polystyrene-based materials, achieving adsorption equilibrium within 10 min, but also demonstrates excellent comprehensive performance. The resin exhibits an adsorption capacity of up to 141.37 mg·g^−1^ for Sr^2+^ at pH 6 and remains stable under a gamma radiation dose of 500 kGy. Furthermore, density functional theory (DFT) calculations provided molecular-level insights into the adsorption process by revealing a synergistic coordination mechanism. Comparative studies by Song [73] and Liu [74] indicate that chemically bonded materials exhibit superior adsorption kinetics, cyclic stability, and radiation resistance compared to physically impregnated materials. Furthermore, fixed-bed column experiments by Prathibha [75] et al. confirmed that the DtBDCH18C6-coated resin could selectively retain Sr^2+^ from simulated fast reactor HLLW containing 59 elements, and the feasibility of its large-scale application was validated via the Thomas model. Although a portion of Ba^2+^ may be retained on the resin column due to its ionic radius being similar to that of Sr, its interference can be effectively minimized by optimizing the elution procedure.

In addition to the chemical bonding strategy, the physicochemical properties of the support matrix, such as pore structure, specific surface area, and morphology, play a decisive role in determining the column efficiency, mass transfer kinetics, and practical applicability of the functional materials. Silica gel is considered an ideal chromatographic support due to its high mechanical strength, acid–base resistance, and large specific surface area. Xu and Zhang et al. utilized macroporous SiO_2_-P supports to impregnate N,N,N′,N′-tetraoctyl diglycolamide (TODGA) or crown ethers, and the resulting adsorbents exhibited high affinity for Sr^2+^ or Y^3+^ in nitric acid media [76,77]. Momen et al. compared polysulfone (PS) microcapsules with commercial Sr-resin and found that the microcapsules, characterized by dense internal pore structures and uniform extractant distribution, demonstrated significantly superior theoretical plate numbers compared to the traditional resin [78]. Regarding novel morphological materials, Horita et al. developed crown ether-functionalized adsorbent fibers. By employing emulsion graft polymerization to confine adsorption sites to the fiber surface, they drastically shortened the diffusion path [79]. These fibers reached adsorption equilibrium within 10 s, a rate over 100 times faster than commercial resins, effectively resolving the slow kinetics caused by traditional micropore diffusion limitations. Additionally, research has explored crown ether-coated magnetic microspheres, aiming to achieve rapid solid–liquid separation using external magnetic fields [80].

### 2.3. EXC Resins Containing Amide Groups

**Figure 4:**
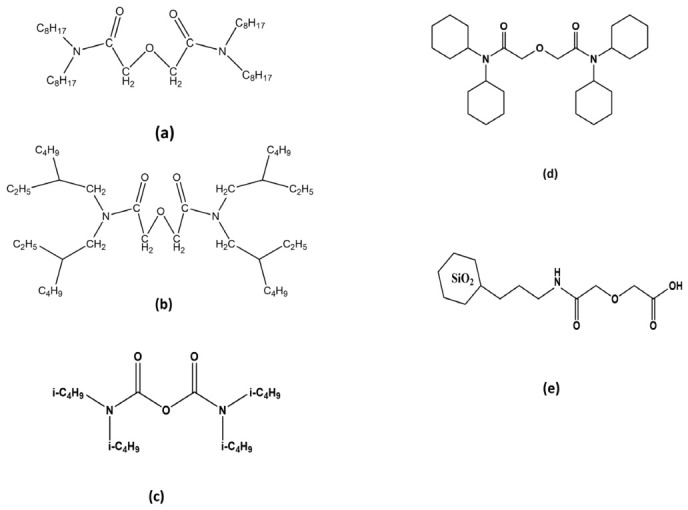
Structural formula of the extractant: (**a**) TODGA; (**b**) T2EHDGA; (**c**) N,N,N′,N′-tetraisobutyl-diglycolamide (TiBDGA); (**d**) N,N,N′,N′-tetracyclohexyldiglycolamide (TCHDGA); (**e**) the diglycolamic acid-grafted silica adsorbent (DGAA-Si).

DGA based extractants have garnered significant attention due to their high structural tunability and excellent separation selectivity. These ligands tend to form stable trigonal chelating complexes with metal ions via two carbonyl oxygen atoms and one ether oxygen atom, most of which shows exceptional extraction performance towards trivalent lanthanides and actinides [81]. There is substantial evidence demonstrating that DGA ligands, such as TODGA [10] (Figure 4a) and N,N,N′,N′-tetra-2-ethylhexyldiglycolamide (T2EHDGA) [8] (Figure 4b), are capable of separating Y from Sr. Dutta et al. reported an extraction chromatographic resin based on TODGA impregnated onto Chromosorb W inert support for the separation of NCA ^90^Y from ^90^Sr [10]. Studies have shown that in a 4 M HNO_3_ medium, Y^3+^ exhibits a high distribution ratio while Sr^2+^ is barely adsorbed, thereby achieving effective separation of the two elements. By eluting ^90^Sr with 0.01 M HNO_3_ followed by the selective elution of ^90^Y using 0.01 M EDTA solution at pH 2.0, high-purity ^90^Y verified by half-life determination can be obtained, meeting the requirements for radiopharmaceutical preparation. However, the resin suffers from performance degradation upon repeated use and limited radiation stability, indicating that its immobilization strategy still requires further optimization. Wu et al. developed a porous silica-based adsorbent, (TODGA + 1-dodecanol)/SiO_2_-P-F600, for the selective separation of Y^3+^ from Sr^2+^ in acidic solutions. The study demonstrated that the impregnated adsorbent exhibited significantly higher affinity towards Y^3+^ compared to Sr^2+^ across various acid concentrations. Thermodynamic analysis confirmed the process was spontaneous and exothermic. Successful chromatographic separation was achieved using a packed column. In the HNO_3_ system, Y^3+^ was selectively recovered with a 96.85% yield using a 0.01 M DTPA eluent, whereas in the HCl system, a 0.1 M HCl solution was sufficient for Y^3+^ elution [82].

Beyond impregnated extraction chromatographic resins (XCR), another pivotal strategy involves the covalent immobilization of extractants. For instance, polymer-grafted resins, such as Levextrel-type resins, are synthesized by covalently bonding DGA ligands onto PS-DVB skeletons. Studies have shown that resins containing TiBDGA (Figure 4c) exhibit a K_d,Sr_ as high as 7300 in 2 M HNO_3_, with negligible adsorption for Rb^+^, demonstrating exceptional selectivity for alkaline earth metals [83]. Although this research focused on Sr-Rb separation, its synthetic strategy provides a valuable template for constructing Y/Sr selective resins. Alternatively, silica-supported adsorbents are fabricated by grafting DGA or its derivatives onto silica surfaces via silane coupling reactions. The DGAA-Si (Figure 4e), developed by Kim et al., enables the gradient elution of Y^3+^ and Sr^2+^ across varying acid concentrations, confirming the feasibility of immobilized DGA systems for chromatographic separation [84].

Mohapatra et al. evaluated a series of C4DGA ligands functionalized with four to eight DGA arms [85]. Their study demonstrated that these ligands achieved highly efficient extraction of Y^3+^ with negligible uptake of Sr^2+^ in 3 M HNO_3_, yielding exceptionally high SF_Y/Sr_ in the range of 10^5^–10^6^. Subsequent research employing a flat-sheet SLM system further validated this selectivity, achieving the transport of over 97% of Y^3+^ within 6 h, while the migration of Sr^2+^ remained negligible [86].

While the aforementioned studies primarily focus on the separation of Y^3+^ from Sr^2+^, parallel research efforts have been devoted to modifying DGA ligands for the selective extraction of Sr^2+^ itself. Li et al. systematically investigated a series of cyclohexyl-substituted DGA ligands and found that, unlike TODGA, which preferentially extracts Y^3+^, the introduction of cyclohexyl groups significantly enhanced Sr^2+^ extraction. In 2.0 M HNO_3_, the distribution ratios of Sr^2+^ increased by a factor of 8–80 relative to conventional TODGA [87]. Subsequent studies by Li et al. [88] and Peng et al. [89] demonstrated that TCHDGA in o-nitrophenyl hexyl ether (NPHE) exhibited Sr^2+^ extraction efficiencies approximately 170 times higher than those of TODGA. Mechanistic investigations revealed the formation of stable 1:3 Sr^2+^-TCHDGA complexes, and simulated HLLW experiments achieved Sr^2+^ recoveries exceeding 99.9% with decontamination factors of up to three orders of magnitude for coexisting ions [88,89].

Although these studies were originally developed for Sr recovery from HLLW rather than ^90^Sr-^90^Y generator applications, their findings remain relevant to radiopharmaceutical production. The radionuclidic purity of the parent ^90^Sr feed solution directly influences the quality of the subsequently generated ^90^Y product. Therefore, highly selective Sr^2+^ extractants such as TCHDGA could potentially be employed as feed-purification or polishing materials to remove competing ions prior to the primary Y-selective separation step. Although these studies were originally developed for Sr recovery from HLLW rather than ^90^Sr-^90^Y generator applications, their findings remain relevant to radiopharmaceutical production. The radionuclidic purity of the parent ^90^Sr feed solution directly influences the quality of the subsequently generated ^90^Y product. Therefore, highly selective Sr^2+^ extractants such as TCHDGA could potentially be employed as feed-purification or polishing materials to remove competing ions prior to the primary Y-selective separation step. Moreover, the pronounced preference of these ligands for Sr^2+^ over competing metal ions suggests that they may also serve as promising candidates for Sr-retention chromatographic systems, in which the parent radionuclide is selectively retained while ^90^Y is recovered in the eluate. In this context, the exceptional Sr^2+^ selectivity demonstrated by TCHDGA-based systems provides valuable design principles for the development of next-generation ^90^Sr-^90^Y generator materials.

In parallel, the use of non-traditional diluents has also been explored to enhance Sr^2+^ extraction. Ma et al. reported that the DGA ligand DPDBDGA exhibited excellent Sr^2+^ extraction performance in the ionic liquid [C_4_mim][NTf_2_], enabling quantitative recovery from dilute nitric acid solutions [81]. Similarly, Xu et al. found that the distribution ratio of Sr^2+^ for DMDODGA in [C_6_mim][Tf_2_N] was approximately two orders of magnitude higher than that obtained in conventional octanol/kerosene systems [90]. These results demonstrate that ionic liquids can substantially enhance Sr^2+^ extraction by altering the extraction mechanism. Nevertheless, despite the promising extraction performance of these modified DGA systems, their implementation in practical chromatographic platforms for routine ^90^Sr-^90^Y generator operation has not yet been demonstrated, representing an important direction for future research.

As summarized in, the extraction chromatographic materials reported for the separation of ^90^Sr and ^90^Y exhibit significant differences in separation mechanisms, selectivity, and technological maturity. Overall, HDEHP-based materials remain the most technologically mature and widely applied separation systems. These materials have been successfully validated under radioactive conditions ranging from mCi to Ci levels, demonstrating excellent engineering feasibility, operational stability, and scalability. In particular, the HDEHP-CMPO composite system has been validated at the 5 Ci level while maintaining a ^90^Sr breakthrough below 10^−8^, representing the highest degree of engineering demonstration reported among the currently available extraction chromatographic materials.

In contrast, crown ether-based materials employ a fundamentally different separation strategy, selectively retaining Sr^2+^ while allowing Y^3+^ to pass through the chromatographic column. Through highly specific host-guest recognition, these materials exhibit exceptional affinity toward Sr^2+^, typically achieving Sr/Y separation factors exceeding 10^3^–10^4^ and quantitative or near-quantitative recovery of ^90^Y. Such characteristics make crown ether-based materials highly promising candidates for next-generation ^90^Sr-^90^Y generators. However, compared with HDEHP-based systems, their applications have largely remained at the laboratory or pilot scale, and comprehensive evaluations of long-term operational stability and large-scale radiological performance are still limited.

DGA-based materials exhibit remarkably high affinity toward Y^3+^ and excellent separation performance. In some studies, Y/Sr separation factors as high as 10^5^ have been reported, while Y recovery consistently exceeds 90%. From the perspective of separation efficiency, DGA-based materials have demonstrated clear advantages over conventional systems. Nevertheless, most investigations remain limited to laboratory-scale evaluations, and systematic studies on long-term cycling performance, radiation resistance, and continuous column operation are still lacking.

Overall, a comparison of the performance and application status of the materials summarized in Table 1 indicates that HDEHP based systems remain the most technologically mature extraction chromatographic materials for ^90^Sr-^90^Y separation, owing to their well-established process routes, reliable radiological validation, and demonstrated scalability. In contrast, crown ether- and DGA-based materials provide alternative separation strategies and show considerable potential for future development, although further studies on large-scale operation, long-term stability, and radiological validation are still required.

## 3. Ion Exchange Resins and Stationary Phases

### 3.1. Organic Ion Exchange Materials

Strong acid cation exchange resins, featuring sulfonic acid groups (-SO_3_H) as their functional moieties, adsorb cations via electrostatic interactions in acidic media. This type of resin precisely exploits the distinct differences in ionic radius, charge density, and hydration energy between Sr^2+^ and Y^3+^, thereby achieving highly selective separation and purification of the two ions. Studies by Muchtaridi et al. revealed that in a 6 M HCl medium, despite the higher charge of Y^3+^, its larger hydrated ionic radius compared to Sr^2+^ results in weaker electrostatic interactions with sulfonic acid groups, thereby facilitating the preferential elution of Y^3+^. It appears that hydration energy and ionic volume are frequently more influential than the bare ionic charge in determining separation outcomes [91]. However, conventional resins typically possess a narrow selectivity window. Studies by Vasylyeva et al. indicated that as solution concentration increases, the selectivity of Dowex HCR-s/s resin for Sr, Y, and Zr decreases significantly, often necessitating the use of specific complexing eluents such as EDTA to achieve separation. Furthermore, work by Grahek et al. confirmed that although alcohol–water mixed systems can remove substantial amounts of matrix ions, the overly strong binding of Sr and Y to the resin results in sluggish elution kinetics and susceptibility to interference from other lanthanides, thereby limiting their direct application in complex matrices [92]. To overcome these limitations, an alternative strategy employs complexing agents to reverse the elution order on sulfonic acid resins. Complexing agents such as trisodium phosphate or DCTA are utilized to form stable anionic complexes with Y^3+^, while Sr^2+^ remains in a free cationic state. Studies by Kumar, Xu, and others have validated the high efficiency of this strategy in both environmental sample analysis and the purification of medical radionuclides [93,94].

The carboxyl group (-COOH), functioning as a weak acidic moiety, exhibits pH dependent proton dissociation and typically demonstrates a higher affinity for high-valence metal ions. Consequently, it serves as an ideal candidate for Sr-Y separation. In 2025, Shi et al. developed a novel carboxyl-functionalized ion-exchange resin specifically designed for the efficient separation of trace ^90^Y from simulated ^90^Sr decay systems [95]. This resin exhibits excellent selectivity for Y^3+^ within complex matrices, providing a new material platform for the purification of medical-grade ^90^Y. Similarly, Zhou et al. reported a polyhydroxamic acid (PHA) resin prepared via γ-irradiation modification of polyacrylamide. In acetate or citrate buffer systems, this resin facilitates the ternary separation of Zr^4+^/Y^3+^/Sr^2+^, achieving an elution efficiency of 99.9% for Y^3+^ [96].

In strongly acidic media such as nitric, hydrochloric, or sulfuric acid, Y^3+^ is capable of forming stable anionic complexes with inorganic anions, whereas alkaline earth metal ions like Sr^2+^ typically do not form such complexes. This difference in chemical behavior provides a critical pathway for the separation of Y from alkaline earth metals and complex matrices using anion exchange resins. As early as 1961, Misumi et al. successfully achieved the separation of radioactive ^90^Y from ^90^Sr using a carbonate-form strong base anion exchange resin (Dowex-1) in an ammonium carbonate solution. Their study revealed that ^90^Y was firmly adsorbed onto the resin in the form of an anionic complex, whereas ^90^Sr remained unadsorbed and eluted directly with the effluent, thereby enabling the efficient recovery of NCA ^90^Y. This foundational work established that precisely tailoring the medium environment to induce the formation of Y anionic complexes is an effective strategy for selective separation from alkaline earth metals and other complex matrix elements [97].

El-Shahawi et al. proposed a facile separation strategy based on ammonium nitrate-modified Chelex-100 adsorbent. This method exploits the selective retention capability of the modified adsorbent for Y^3+^ at pH 1.0 to achieve effective separation from Sr^2+^, which passes directly through the chromatography column. Studies indicate that the adsorption kinetics of Y^3+^ are rapid, reaching equilibrium within 40 min and conforming to the pseudo-first-order kinetic model. The process offers advantages such as low cost, operational simplicity, and high radiochemical purity of the eluate, providing a viable pathway for rapid laboratory-scale separation. However, the selectivity of this method may face challenges when treating complex industrial wastewater containing high concentrations of competing ions [98]. To address the demand for extracting ^90^Sr from complex matrices, such as irradiated nuclear fuel dissolutions, and for developing ^90^Sr-^90^Y generators, Sankar et al. developed an integrated process combining crown ether extraction with ion exchange chromatography. The study first utilized DtBuCH18C6 to efficiently extract ^90^Sr from mixed carbide fuel dissolutions, subsequently immobilizing it on Dowex 50WX8 cation exchange resin. By employing sodium trimetaphosphate (SMP) as an eluent, ^90^Y could be periodically and selectively eluted. The prepared ^90^YCl_3_ met therapeutic-grade radiopharmaceutical standards, being free from α, β, and γ impurities. Furthermore, each gram of irradiated fuel yielded approximately 7.7 GBq of ^90^Y, which is sufficient to meet clinical therapeutic dosage requirements. Although this method demonstrates excellent performance in terms of purity and yield, it relies on expensive crown ether extractants and complex nuclear fuel reprocessing environments. Additionally, the phosphates present in the eluent may increase the purification burden prior to subsequent drug labeling [99]. Overall, these studies demonstrate that both speciation control and hybrid separation schemes are essential for achieving high-purity ^90^Y in generator systems.

As summarized in Table 2, the currently reported organic ion-exchange materials for Sr/Y separation mainly rely on selective coordination of Y^3+^ by oxygen-containing functional groups or on differences in ionic speciation under controlled chemical environments. Carboxyl- and hydroxamate-based resins generally exhibit excellent affinity toward Y^3+^, achieving Y recoveries above 99%, while the SiAaC resin displays the highest reported selectivity with a KF_Y/Sr_ value of 9900. Anion-exchange systems, represented by Dowex-1, utilize the formation of anionic yttrium complexes to achieve efficient separation from Sr^2+^, whereas cation-exchange systems such as Dowex 50 W × 8 combine ion-exchange and complexation strategies to enable repeated ^90^Y elution from retained ^90^Sr. Notably, the Dowex 50 W × 8 process has been demonstrated under radioactive conditions and achieved a production capacity of 7.7 GBq of ^90^Y per gram of irradiated fuel, highlighting its practical applicability. Nevertheless, compared with extraction chromatographic systems, the number of organic ion-exchange materials specifically developed for ^90^Sr-^90^Y generators remains limited, and comprehensive evaluations of radiation stability, long-term cycling performance, and large-scale operation are still lacking.

### 3.2. Inorganic Ion Exchange Materials

#### 3.2.1. Antimony-Based Inorganic Ion Exchangers

Antimony-based inorganic ion exchangers, particularly functional materials centered on antimony pentoxide (Sb_2_O_5_) and its hydrate, polyantimonic acid (PAA), have emerged as a research hotspot in Sr-Y separation. This prominence is attributed to their unique affinity for Sr^2+^, exceptional acid stability, and tunable surface microenvironments [100].

Beginning in the 1970s and extending into the 1980s, Abe and colleagues conducted systematic investigations into the ion-exchange properties of crystalline antimonic(V) acid (C-SbA) with respect to alkaline earth metals. A key observation was C-SbA’s unusual selectivity sequence for trace alkaline earth metals in nitric acid media: Mg^2+^ < Ba^2+^ < Ca^2+^ < Sr^2+^ [101]. Recent studies attribute the selectivity mechanism for Sr-Y separation partly to differences in charge density and coordination requirements. DFT calculations indicate that the interaction between Sr^2+^ and Sb-OH groups is dominated by ion exchange, accompanied by charge transfer and a reduction in system energy. The material’s inherent porous structure and channel characteristics allow Sr^2+^ to effectively access the lattice tunnels or surface sites of the antimonic acid. While the specific coordination behavior of Y^3+^ and the exact impact of proton competition under highly acidic conditions are not explicitly detailed in the DFT analysis, experimental results show that Sb_2_O_5_/SiO_2_ exhibits good selectivity for Sr over Y, suggesting that Y remains largely in solution during the selective adsorption of Sr [102]. In addition to the aforementioned mechanisms, the compatibility between ion size and the material’s pore structure, the difference in hydration energy between Sr^2+^ and Y^3+^, and the variation in adsorbent surface charge across varying pH conditions collectively influence this selective separation performance [102,103,104,105].

To overcome the inherent limitations of traditional antimonic acid materials, such as sluggish kinetics and poor mechanical strength, researchers have actively developed various composite modification strategies to synergistically enhance their overall performance. Among these, silica-based support composites have achieved significant optimization by ingeniously combining active components with porous frameworks. Loading active Sb_2_O_5_ components onto the surface of porous SiO_2_ microspheres not only substantially improves mechanical stability but also enhances mass transfer rates by shortening ion diffusion pathways. The Sb_2_O_5_/SiO_2_ adsorbent prepared by Zhang’s team using a vacuum impregnation-oxidation process exhibited excellent kinetic performance. The adsorption equilibrium of Sr^2+^ was reached within just 5 min at pH 6, with a high adsorption capacity of 160.6 mg·g^−1^. Even in a 1 M HNO_3_ strong acid environment, the adsorption capacity remained stable at 51.8 mg·g^−1^ after 30 min. Notably, this material demonstrated exceptional Sr-Y separation selectivity at pH 6, with an SF_Sr/F_ exceeding 7769 and Y purity reaching 99.7% in column experiments [102]. It should be noted, however, that this purity, corresponding to approximately 0.3% residual Sr, remains substantially below the radiochemical purity threshold required for medical-grade ^90^Y production, where ^90^Sr impurities must be reduced to below 2 × 10^−3^% of total activity as recommended by ICRP [13]. Similarly, the PAA-XAD composite, prepared by Sivaiah’s team through in situ precipitation of PAA onto Amberlite XAD-7 macroporous resin, also achieved a performance breakthrough. It demonstrated a Sr adsorption capacity of 23 mg/g and a high K_d_ of 6069 mL·g^−1^ in 0.1 M HNO_3_, providing a new and efficient material system for Sr-Y separation [106].

Building upon these composite strategies, recent advancements have focused on integrating phosphatoantimonates with polymer matrices to address practical application challenges. For instance, Li et al. successfully developed K_2_SbPO_6_/polyacrylonitrile (PAN) composite microspheres through an automated peristaltic pump titration method [107]. This innovative approach not only facilitates the mechanized preparation of uniform microspheres, effectively overcoming the solid–liquid separation difficulties associated with powdered materials, but also yields a material with remarkable performance. The K_2_SbPO_6_/PAN microspheres exhibit a high maximum adsorption capacity of 131.15 mg·g^−1^ for Sr^2+^, maintain a high removal rate (R_Sr_ > 90%) across a wide pH range (pH 3–12), and demonstrate excellent radiation resistance. Furthermore, column adsorption experiments confirmed their capability for efficient dynamic capture of Sr^2+^ ions, even at high bed volumes (R_Sr_ > 81% at 970 bed volumes), highlighting their potential for continuous treatment of Sr^2+^-containing solutions. Beyond oxide-based composites, novel metal sulfide composites incorporating antimony have also shown promise. Liu et al. [108] reported a MnS_2_-Sb_2_S_3_ (MSC) composite that achieved ultra-high selectivity for Y^3+^ over Sr^2+^, with an SF_Y/Sr_ as high as 6.27 × 10^5^. This material exhibits rapid adsorption kinetics, reaching equilibrium within 5 min, excellent pH stability (pH 2.03–11.95), and reusability, making it a promising candidate for medical ^90^Y generators. However, even with improved stability, some metal sulfide composites still show limitations, such as decomposition and color change at pH 1, indicating that structural integrity under extremely harsh acidic conditions remains a challenge [108]. The ion exchange mechanism between Y^3+^ and Mn^2+^, facilitated by their similar ionic radii, underpins this high selectivity.

Metal doping stands as a pivotal strategy for regulating and enhancing the performance of antimony-based materials, with its modification mechanism primarily rooted in the dual principles of structural geometric and surface electronic effects. From a structural geometric perspective, the ionic radius of the dopant plays a critical role in determining the material’s skeletal stability. Studies consistently show that introducing dopants with ionic radii similar or comparable to antimony, such as silicon and vanadium, is crucial for maintaining the integrity of the pyrochlore structure, thereby preserving efficient ion exchange channels. For instance, silicon-doped SiSb-0.5 and vanadium-doped VSb-0.5 antimony oxides, thanks to their stable structures, demonstrate impressive adsorption capacities for Sr^2+^ in 3 M HNO_3_ strong acid media, achieving 44.30 mg·g^−1^ and 38.93 mg·g^−1^, respectively. Conversely, an unsuitable ionic radius or excessively high doping concentrations can lead to detrimental lattice distortion or even structural collapse, ultimately diminishing adsorption performance [104]. Beyond structural considerations, dopants exert unique surface electronic effects, subtly modulating the material’s surface electronegativity and the abundance of hydroxyl sites. This optimized surface characteristic is instrumental in promoting the generation of cation exchange sites in strong acid environments, which in turn significantly boosts the material’s K_d_. A compelling illustration of this is provided by Huang et al., whose lanthanum doping strategy successfully engineered the surface charge distribution and hydroxyl group density on Sb_2_O_5_, leading to a remarkable improvement in Sr-Y separation efficiency under harsh acidic conditions [100]. This exemplifies the power of surface property modulation in practical applications. Furthermore, dopants with specific valence states can impart highly specialized recognition capabilities. A notable example is the incorporation of W^6+^, which not only successfully preserves the pyrochlore structure of the host material but also dramatically enhances its adsorption selectivity for Cs [109]. In a related development, Zhao et al. introduced a pH-controlled potassium thioantimonate (SbS-1K) that demonstrates switchable coadsorption and separation capabilities for mixed Cs^+^ and Sr^2+^. This material exhibits ultrafast kinetics and high removal rates for both ions at pH 6, but selectively inhibits Sr^2+^ adsorption at pH 2, achieving a high SF_CS/Sr_ of 256. However, even for optimized materials like SbS-1K, structural stability can still be challenged under extremely harsh acidic conditions as 3 M HCl, potentially leading to dissolution or decomposition [110]. Despite these advancements, the selectivity of antimony-based materials still requires further optimization in complex actual waste liquids containing high concentrations of competing ions (e.g., Na^+^, K^+^, Mg^2+^, Ca^2+^), as their adsorption performance for target ions can be inhibited, leading to a decrease in K_d_ [107,110]. Such findings underscore the vast potential of doping strategies in developing multifunctional, highly selective materials for radionuclide separation.

Ultimately, while composite and doping strategies have significantly improved performance, critical questions remain regarding the low-cost, large-scale production and long-term reliability of these materials in actual nuclear waste treatment environments, including their radiation resistance and regeneration capacity.

#### 3.2.2. Manganese Oxide

In ^90^Sr-^90^Y generator systems, manganese oxide-based materials operate through two complementary separation strategies including the selective retention of Y^3+^ for parent–daughter separation and the preferential immobilization of Sr^2+^ for ^90^Y purification. These separation behaviors arise from the layered or tunnel structures characteristic of manganese oxides, and are fundamentally distinct from simple single-ion adsorption processes Although both strategies exploit the layered or tunnel structures characteristic of manganese oxides, their underlying chemical logic and operational principles differ substantially: the former relies on the affinity of MnO_2_ surfaces for hydrolyzed Y species, while the latter leverages the high charge density of interlayer sites for Sr^2+^ exchange [13,111,112].

Hydrated manganese dioxide (HMD) is the representative material of the “Y adsorption/Sr elution” strategy in early ^90^Sr-^90^Y generator research. The core advantage of this system lies in the pronounced difference in adsorption behavior between Y^3+^ and Sr^2+^ under specific acidity conditions. In 0.001–0.1 M HNO_3_, HMD strongly adsorbs Y^3+^ (recovery exceeding 90%) while showing negligible adsorption toward Sr^2+^, enabling efficient preliminary separation. Subsequently, the retained Y^3+^ can be quantitatively eluted with 1 M HNO_3_, yielding a high-purity ^90^Y solution. Process optimization established an optimal column height-to-diameter ratio of 5:1 to balance flow rate and separation efficiency [112,113]. Despite the favorable selectivity demonstrated in static adsorption experiments, the long-term reliability of HMD as a separation medium is constrained by structural stability issues. Dynamic exchange capacity attenuates during repeated adsorption–elution cycles. Although the material tolerates common ions, separation selectivity may be compromised in complex matrices containing complexing agents or organic matter. Furthermore, current preparation processes face challenges in reproducibility and scalability [112,113].

In contrast to HMD, layered birnessite and its derivatives exhibit high selectivity for Sr^2+^, positioning them as materials suited for the purification of ^90^Y. Chakravarty et al. systematically demonstrated that synthetic sodium birnessite (Na_0.55_Mn_2_O_4_·1.5H_2_O) in an equilibrated ^90^Sr-9^0^Y mixture efficiently adsorbs the parent ^90^Sr, enabling ^90^Y to be recovered with a separation yield exceeding 80% [13]. The ^90^Sr impurity in the resulting ^90^Y was below 1 × 10^−4^%, significantly exceeding the ICRP standard of 2 × 10^−3^%, and the purified ^90^Y was successfully used to prepare a melanoma-targeting radiopharmaceutical. However, the separation efficiency of the birnessite system is critically dependent on the stability of its layered structure; competing ions such as Ca^2+^ and organic complexing agents may reduce selectivity for Sr^2+^, thereby compromising ^90^Y purity. Challenges in dynamic adsorption, mechanical stability, and regeneration efficiency remain barriers to scale-up.

It is crucial to differentiate these Sr/Y separation studies from the broader literature on manganese oxide-based Sr^2+^ adsorption. Extensive research has focused on modifying manganese oxide through various complementary approaches. Researchers have introduced metal-ion doping with elements like Fe, Co, and Ni to suppress Jahn–Teller distortion and improve cycling stability [114]. Other strategies include Zr coprecipitation to extend the operational pH range into acidic conditions [115] and the use of organic short-peptide templating to enhance dispersibility and specific surface area [116]. Furthermore, morphology engineering has produced mesoporous hexagonal birnessite [117], flower-like MnO_2_ [118], tunnel-structured MnO_2_ [119], and nanowire MnO_2_ [120]. The resulting pore architecture plays a strong role in influencing adsorption kinetics [121]. Together, these studies have significantly advanced Sr^2+^ adsorption capacity, cycling durability, and tolerance to competing ions.

Despite these advances, none of the doping or morphology-engineering studies have evaluated selectivity between Sr^2+^ and Y^3+^ in an equilibrated ^90^Sr-^90^Y mixture. Their direct contribution to generator technology therefore remains unestablished. It is currently an open question whether the observed gains in capacity and stability actually translate into high ^90^Y purity or separation yield. Bridging this gap requires a systematic evaluation of these advanced materials in binary Sr/Y systems. Future work must provide quantitative reporting on decontamination factors, ^90^Sr breakthrough levels, and elution profiles. These metrics are essential for assessing generator viability but are currently absent from the literature.

#### 3.2.3. Zeolite-like Molecular Sieves

Zeolites are a class of crystalline aluminosilicates defined by an ordered microporous framework constructed from corner-sharing silicon and aluminum-oxygen tetrahedra [122]. This architecture generates specific three-dimensional channels and cages that accommodate exchangeable cations like Na, K, and Ca, alongside water molecules [123,124]. These structural features collectively endow zeolites with superior ion-exchange capacity, high specific surface area, and exceptional thermal and chemical stability.

For Sr^2+^, the adsorption process is primarily governed by a classical ion-exchange mechanism. Studies indicate that CHA-type zeolites can efficiently capture Sr^2+^ via rapid ion exchange across a broad pH range of 3–11, while maintaining substantial adsorption capacity even after five regeneration cycles [125]. Similarly, synthetic 4A zeolite exhibits the K_d_ as high as 1298 mL·g^−1^ in simulated HLLW, confirming the high affinity of the LTA structure toward Sr^2+^ [126]. In contrast, the adsorption mechanism of Y^3+^ is markedly more complex. Crystallochemical investigations by Guzzinati [127] et al. revealed that Y^3+^ exhibits distinct site selectivity within FAU-type zeolites, preferentially occupying the spatially confined β-cages and forming stable octahedral coordination with framework oxygen atoms. Notably, the adsorption behavior of Y^3+^ does not follow the conventional ion-radius-dependent trend. Its adsorption affinity on NaX zeolite is approximately twice that of Nd^3+^, and it does not induce significant framework dealumination or the formation of supramolecular clusters as observed with Nd^3+^. This divergence in adsorption mechanisms provides a theoretical basis for the highly selective separation of Sr-Y using zeolite-based materials.

To further optimize separation performance, significant strides have been made in the synthesis strategies and modification engineering of zeolites. On one hand, the synthesis of 4A zeolite (Z4A) utilizing Bayer process waste not only facilitates the resource utilization of industrial solid waste but also demonstrates Sr removal efficiency in simulated seawater, achieving an 84% removal rate within just 5 min, thereby validating the feasibility of cost-effective synthesis routes [128]. On the other hand, precise modulation of topological structures is equally critical. K-F type zeolite (chabazite structure) exhibits superior ion-sieving effects in Cs^+^/Sr^2+^ co-existing systems, with a Sr^2+^ removal rate as high as 98% while showing negligible adsorption for Cs^+^ [129]. Furthermore, to address the bottleneck of insufficient selectivity in natural zeolites, the design of composite materials has emerged as a research hotspot. For instance, loading CuO nanoparticles onto Ag-clinoptilolite significantly improves the adsorption kinetics for ^90^Sr [130]. Meanwhile, the Zeolite A/Calcium-deficient Hydroxyapatite (Ca-def HA) nanocomposite developed by Watanabe [131] et al. effectively suppresses Sr^2+^ leaching under alkaline conditions via a surface coating mechanism, thereby enhancing the material’s long-term stability.

From an engineering perspective, the regeneration of adsorbents and the final disposal of saturated materials are of paramount importance. Studies indicate that the TiO_2_-NaA@PAN composite membrane retains 81.6% of its adsorption efficiency after three elution cycles using sodium citrate. Meanwhile, EDTA is widely employed for the quantitative elution of Sr due to its ability to form stable complexes with the metal ion [132,133]. In response to complex multi-ion competitive environments, the thiol-functionalized NaA zeolite developed by Li [134] et al. and the sulfur-encapsulated NaA zeolite engineered by Yang [135] et al. both achieve highly selective capture of Sr^2+^ while maintaining excellent structural stability, by introducing soft acid–soft base interaction mechanisms. Furthermore, addressing complex effluent environments, the TiO_2_-NaA@PAN membrane developed by Liu et al. and the cost-effective zeolite materials by Fotsop et al. have both demonstrated robust stability in multi-ion competitive systems, providing valuable references for practical wastewater treatment engineering [136].

In response to the strong complexation characteristics of Y^3+^, researchers have developed various functionalized separation systems. Based on the principle of extraction chromatography, HDEHP/SiO_2_-P resin provides a viable strategy for preparing zeolite materials that specifically adsorb Y^3+^. This involves modifying zeolites with organic extractants to enhance their adsorption capacity [76,82]. Additionally, Zhang et al. proposed an innovative “electrolysis–high temperature molecular sieve adsorption” combined strategy. At 773 K, using 5A molecular sieves increased the removal rate of Y^3+^ in molten salt from 93.11% to 99.94%, expanding the application of zeolites in high-temperature molten salt systems [137]. For mixed ion systems, Mimura et al. confirmed that constructing mixed zeolite columns such as Y-type + natural mordenite, can achieve gradient separation of Sr and Cs, providing an engineering example for the synergistic removal of multiple radionuclides [138,139].

Although zeolite materials have demonstrated significant potential for Sr^2+^ adsorption, their application in the efficient separation of Sr-Y systems faces numerous formidable challenges. Conventional zeolites generally exhibit weak adsorption affinity for Y^3+^, often necessitating modification with organic extractants to enhance affinity. However, organic phases pose a risk of degradation under strong acidic and high-radiation conditions. In real radioactive waste streams characterized by high salinity and multi-component complexity, the identification and capture of target ions are severely impeded by the presence of abundant competing ions. In summary, current research remains largely focused on the separation of single Sr^2+^, and there is a distinct lack of rationally designed zeolite architectures specifically for the efficient separation of Sr-Y systems, which has become a critical bottleneck restricting the development of this technology.

Moving forward, breakthroughs in this field will rely on four key directions. First, rather than simply repurposing existing frameworks, the development of novel zeolite topologies is essential to achieve high separation factors for the Sr-Y system. Second, to enhance selectivity, researchers can leverage the confined spaces within zeolite pores to anchor functional groups, such as carboxyl or phosphonate moieties, thereby constructing active sites with specific coordination for Y^3+^. Third, the integration of machine learning algorithms offers a promising avenue to predict framework-ion interactions, accelerating the screening process beyond traditional trial-and-error approaches. Finally, from an engineering perspective, the application of zeolite membrane technology in continuous separation processes could be advanced. Although LTA membranes have already demonstrated a Sr^2+^ rejection rate exceeding 99% [140], their potential within the Sr-Y separation system remains to be fully explored.

#### 3.2.4. Other Inorganic Ion Exchange Materials

In addition to antimonates, manganese oxides, and traditional zeolites, a series of novel inorganic ion exchangers have been explored for Sr-Y separation systems. The research by Sylvester not only verified the feasibility of using clinoptilolite, potassium titanium silicate, potassium ferrate, sodium titanium silicate, and sodium titanate in developing ^90^Sr-^90^Y generators and obtained patent certification, but also discovered that sodium titanium silicate and sodium titanate exhibit excellent selective adsorption capacity for ^90^Sr with extremely low selectivity for ^90^Y [141], which possess advantages such as radiation resistance, low toxicity, rapid adsorption, and good regenerability, making them promising separation media. However, these materials have not yet undergone testing in high-level radioactive environments, and their practicality in real high-level radioactive waste requires further experimental verification.

Furthermore, although multiple studies have expanded material systems such as functionalized silica [142], cerium iodotungstate [143], sodium titanium silicate [144], lamellar structure silver sulfide nanoparticles [145], zirconium vanadate [146], zirconium titanium phosphate [11] and quinoline phosphomolybdate [147], these works are mostly limited to scenarios involving the separation of NCA ^90^Y from trace amounts of ^90^Sr. There are few experimental reports on separating trace ^90^Y from high-concentration ^90^Sr matrices. In fact, the development of inorganic materials specifically for Sr-Y separation remains relatively scarce. The design intent of most advanced materials is to selectively capture Sr from complex nuclear waste. Current research frontiers have extended to emerging systems such as layered double hydroxides (LDHs) [148,149], titanate salts [150,151], and two-dimensional transition metal carbides/nitrides (MXenes) [152,153]. Although these materials have made progress in the field of Sr^2+^ adsorption, how to achieve precise recognition of Sr^2+^ and Y^3+^ through microstructure regulation, particularly by utilizing the subtle differences in their ionic radii, charge densities, and coordination preferences, remains a key scientific problem that needs to be breakthrough. Future research needs to establish specific separation mechanisms for the Sr-Y pair while strengthening the radiation stability of materials, so as to promote the practical application of ^90^Sr-^90^Y generator technology.

As summarized in Table 3, inorganic ion-exchange materials exhibit diverse separation mechanisms and performance characteristics for Sr/Y separation. Among them, antimony-based materials generally show the highest affinity toward Sr^2+^. The Sb_2_O_5_-SiO_2_ composite achieved an SF_Sr/Y_ exceeding 7.8 × 10^3^and a Y recovery of 99.7%, while maintaining appreciable adsorption capacity even in 1 M HNO_3_. These results highlight the excellent acid stability and separation selectivity of antimony-based adsorbents. Nevertheless, the residual Sr content corresponding to the reported Y purity remains significantly higher than the radiochemical purity requirements for medical-grade ^90^Y, indicating that further optimization is still necessary for generator applications.

Manganese-based materials exhibit complementary separation behaviors. HMD preferentially adsorbs Y^3+^ while allowing Sr^2+^ to pass through, resulting in Y/Sr separation factors exceeding 5 × 10^4^. In contrast, sodium birnessite selectively retains Sr^2+^ and enables the recovery of high-purity ^90^Y from equilibrated ^90^Sr-^90^Y mixtures. Notably, birnessite-based systems have undergone radiological validation at the 185 MBq level and produced ^90^Y with ^90^Sr impurities below the recommended limits, making them among the most promising inorganic materials reported to date. However, their long-term structural stability, regeneration efficiency, and resistance to competing ions remain insufficiently investigated.

Zeolite and titanate-based materials generally demonstrate exceptionally high distribution coefficients for Sr^2+^, frequently reaching 10^4^–10^6^ mL·g^−1^. Their advantages include rapid ion exchange kinetics, radiation resistance, and low toxicity. Nevertheless, most studies focus primarily on selective Sr capture rather than direct Sr/Y separation, and quantitative data regarding Y recovery, separation factors, and generator performance are often unavailable. Consequently, their applicability to practical ^90^Sr-^90^Y generator systems remains difficult to assess.

Other inorganic exchangers, including functionalized silica, silver sulfide, zirconium vanadate, and sodium titanosilicate, have also demonstrated favorable Y recovery and selective adsorption behavior. In particular, MnS_2_-Sb_2_S_3_ exhibited an exceptionally high SF_Y/Sr_ of 6.27 × 10^5^, representing one of the highest selectivities reported among inorganic materials. However, most of these studies remain limited to tracer-scale or laboratory-scale experiments, and comprehensive evaluations under realistic radioactive operating conditions are still lacking.

Overall, inorganic ion exchangers possess several intrinsic advantages over organic materials, including superior radiation resistance, chemical stability, and low cost. Among the currently reported systems, antimony-based materials and manganese oxide-based materials appear to be the most promising candidates for future ^90^Sr-^90^Y generator applications. Nevertheless, systematic studies addressing long-term operation, dynamic column performance, repeated regeneration, and large-scale radiological validation are still required before practical implementation can be achieved.

## 4. Selection and Performance Evaluation of ^90^Sr-^90^Y Generator Materials for Clinical Applications

The transition from idealized simulated solutions to the rigorous demands of clinical practice represents the definitive hurdle for Sr-Y separation technology in the medical field. In the context of therapeutic isotope production, the separation system must operate effectively within complex biological or pharmaceutical matrices, often under stringent conditions requiring high acidity and resistance to intense radiation. These clinical-specific constraints not only dictate the pharmacological efficacy of the final radiopharmaceutical but also impose non-negotiable requirements for patient safety.

Conventional separation methods, while historically significant, are increasingly inadequate for modern clinical applications due to inherent drawbacks including high reagent consumption, the generation of secondary waste, and operational complexity. Therefore, this chapter focuses exclusively on the practical challenges of Sr-Y separation within the medical domain. We will systematically analyze the performance criteria of various materials, aiming to provide a rational basis for selecting high-performance separation systems that ensure both the purity of ^90^Y and the safety of clinical therapies.

### 4.1. Material Selection Criteria

Currently, the majority of medical institutions still rely on commercial channels to procure ^90^Y radiopharmaceuticals. This dependence is primarily constrained by bottlenecks in existing generator technologies regarding eluate chemical forms, radiochemical purity, operational convenience, and long-term stability [2,154]. Therefore, for applications in medical centers or hospital radiopharmacies, the core key to developing efficient and reliable ^90^Sr-^90^Y generators lies in the screening of separation materials and process optimization.

High separation efficiency is paramount. Solid-phase adsorbents must exhibit a significant selectivity difference between ^90^Sr and ^90^Y to ensure the eluted ^90^Y possesses high radiochemical purity while keeping ^90^Sr breakthrough to a negligible level. The ICRP recommends that ^90^Sr impurities in human-administered ^90^Y preparations should be below 2 × 10^−3^% to avoid risks of long-term radiation toxicity [13]. To provide a more quantitative basis for evaluation, clinical and regulatory constraints should be explicitly considered. The ICRP-recommended limit on ^90^Sr content in ^90^Y preparations for human use (2 × 10^−3^% of total activity) provides a regulatory benchmark against which separation performance can be assessed. When combined with knowledge of the Sr/Y activity ratio in the feed solution, this purity requirement can be expressed in terms of the necessary decontamination factor for ^90^Sr, which in turn places a lower bound on the selectivity that the adsorbent must deliver in conjunction with the chosen elution protocol. This translation from regulatory threshold to performance specification allows a given material system to be evaluated against clinical requirements rather than solely against other materials in the literature.

The chemical and irradiation stability of the material directly determines the service life and safety of the ^90^Sr-^90^Y generator. Since the generator must operate repeatedly under strongly acidic conditions, such as 3–8 M HNO_3_ or HCl, and remain exposed to the high-energy beta radiation field of ^90^Sr for extended periods, the material is required to possess dual resistance to strong acid corrosion and radiation degradation. As a long-lived, high-energy beta emitter, ^90^Sr induces continuous radiolysis that degrades organic functional groups or polymer backbones, leading to material performance deterioration. The choice of elution medium also affects separation efficiency, as the ionic strength, pH, and counter-anion identity of the mobile phase all influence the distribution of Sr^2+^ and Y^3+^ between the two phases.

Clinical applications demand the rapid elution of high-concentration ^90^Y to meet therapeutic dosage requirements. Separation materials must therefore exhibit rapid adsorption–desorption kinetics and a sufficiently high saturation adsorption capacity for Y^3+^. Material morphology, whether in the form of particles, membranes, or monoliths, also directly influences column pressure drop, achievable flow rates, and the potential for integration into automated production systems.

The chemical form of the eluted ^90^Y must be inherently compatible with subsequent labeling reactions. Ideally, the ^90^Y should exist as Y^3+^ cations within a mild and biocompatible solution such as an acetate buffer at pH 5.0–5.5. This specific form allows for direct conjugation with chelators like DOTA and DTPA for labeling antibodies, peptides, or microspheres without requiring additional purification or neutralization steps [22,155]. Conversely, elution with strong acids such as 8 M HCl or EDTA necessitates additional neutralization or purification steps prior to DOTA or DTPA bioconjugation, which inevitably increases operational complexity and introduces risks of contamination or reduced radioactive yield [156,157].

The synthesis of separation materials should demonstrate good scalability and controllable raw material costs. Materials should also permit regeneration and reuse over multiple cycles without substantial loss of separation performance, as this directly affects the operational economics and long-term reliability of the generator.

Operational safety and engineering practicality are equally paramount. The design of generators should aim to streamline operational procedures, minimize radiation exposure to personnel. The adsorbent must possess sufficient mechanical strength to withstand repeated fluid pressure cycles during column operation. Certain designs incorporate a dual-column configuration consisting of a primary separation column and a guard column [19,22,158], which ensures that any ^90^Sr escaping the primary column is retained before the final eluate is collected.

### 4.2. Representative Application Cases and Performance Analysis

The ^90^Sr-^90^Y generator is central to producing high-purity ^90^Y for targeted radionuclide therapy. Over the past few years, much work has focused on designing novel adsorbent materials that enable efficient separation of Y^3+^ from Sr^2+^ in concentrated solutions. Below, we summarize key advances from recent reports.

When benchmarked against the clinical requirements outlined in Section 4.1, the surveyed materials fall into three distinct performance tiers, reflecting their proximity to medical-grade ^90^Y production.

Two inorganic material systems have achieved performance levels approaching or meeting the clinical thresholds. Geng et al. developed a PAA-based generator using vacuum freeze-dried PAA with a dual-column configuration and a hold-back carrier technique, in which stable Sr was added to the primary eluate before secondary purification. This approach yielded an SFY/Sr of 10^6^, meeting the medical purity threshold, with a ^90^Y elution efficiency of 77.5% [158]. In a complementary strategy, Zhang et al. constructed a generator based on the MnS_2_-Sb_2_S_3_ (MSC) composite. Using a mild two-step elution protocol (0.2 M NaCl for Sr removal followed by 0.5 M NaHCO_3_ for Y^3+^ elution), they achieved a radionuclidic purity above 99.999% in the ^90^Y product, corresponding to an Sr/Y molar ratio as low as 1:10^9.76^, well beyond medical standards. The MSC material also demonstrated good regenerability over three consecutive cycles without significant loss in crystal structure or separation performance [108]. These two systems also illustrate a practical trade-off: PAA meets the clinical SF benchmark at the cost of a moderate elution yield (77.5%), while MSC delivers both high purity and high recovery but has been tested over three cycles. Neither system, however, has been tested under prolonged ^90^Sr radiation exposure.

A second group of materials has shown considerable selectivity but falls one to two orders of magnitude short of the clinical SF benchmark. Zheng et al. developed SiAaC, a carboxyl-functionalized mesoporous ion-exchange resin synthesized via in situ polymerization. At a Sr/Y molar ratio of 4000:1, the resin exhibited a high adsorption capacity of 119.4 mg·g^−1^ and an SF_Y/Sr_ of 9.9 × 10^3^. Sequential elution with 0.2 M NaCl at pH 3 and 0.1 M HCl yielded over 99% Y recovery with a chemical purity exceeding 99.87% [95]. In the bio-based domain, Chen et al. engineered Salmonella typhimurium VNP20009 to display the lanthanide-binding protein LanM on its surface, encapsulating the bacteria within PEGDA hydrogel beads. This microbial system achieved an SF_Y/Sr_ of 1.1 × 10^5^ and maintained stable separation efficiency over ten adsorption–desorption cycles under irradiation, demonstrating the strongest reusability among all candidates surveyed [159]. Comparing these two systems, SiAaC offers higher adsorption capacity and uses a well-established synthetic resin platform, whereas VNP20009 provides superior selectivity and cyclability through a fundamentally different biological recognition mechanism. Both represent promising platforms that would benefit from further optimization to close the remaining gap to the clinical SF threshold.

Several additional materials have demonstrated basic Y^3+^/Sr^2+^ selectivity but remain at the proof-of-concept stage with respect to medical ^90^Y production. Zheng et al. prepared an HDEHP/SiO_2_-P adsorbent that achieved an SF_Y/Sr_ of 1.93 × 10^3^ at a Sr/Y ratio of 2000:1, with 100% Y recovery and negligible Sr breakthrough in column experiments [160]. Kim et al. reported a DGAA-Si adsorbent prepared via amidation, achieving over 90% Y^3+^ recovery with a Q_max_ of 0.18 mmol·g^−1^; the separation was effective in both HNO_3_ and HCl media, though the SF was not reported [84]. Abdel-Galil et al. employed Aspergillus terreus fungal biomass as a biosorbent, achieving an SF_Y/Sr_ of 1.57 × 10^4^ and a Y^3+^ breakthrough capacity of 63.0 mg·g^−1^ [161]. While these studies collectively confirm that selective Sr-Y separation is achievable across diverse material classes, organophosphorus extractants, DGA-grafted silica, and fungal biomass, their reported SF values (10^3^–10^4^) and the use of near-equimolar Sr/Y feed ratios in some cases indicate that further development is required before clinical applicability can be assessed.

Table 4 summarizes the key parameters and performance metrics of the materials discussed above, together with the corresponding clinical requirements established in Section 4.1.

## 5. Conclusions and Outlook

Despite the fruitful results achieved in laboratory research, Sr-Y separation materials still face numerous limitations and challenges on the path toward clinical practice and industrial application.

First, challenges regarding fabrication processes and scalability arise as the synthesis of many high-performance functional materials, such as certain metal sulfide composites or zeolites with specific topological structures, is currently hindered by complex procedures, low yields, and high costs. Therefore, simplifying preparation protocols to achieve large-scale, highly reproducible industrial production remains the primary obstacle to their commercialization.

Second, long-term stability and radiation resistance present significant hurdles because materials in practical ^90^Sr to ^90^Y generator systems are subjected to harsh acidic and high-energy beta radiation fields. Organic degradation, extractant leaching, and inorganic framework collapse continue to limit service life, while existing validation studies rely heavily on simulations and lack long-term data from real high-radioactivity environments.

Third, in complex matrices, the selectivity limitation becomes critical when treating actual multi-component radioactive waste with high salinity, as widespread competing ions like Ba^2+^, Ca^2+^, and Mg^2+^ severely hinder target ion capture. While many existing materials excel in single-component systems, their separation factor undergoes a sharp reduction under realistic complex conditions.

Fourth, there are certain discrepancies between the experimental conditions reported in the literature and the actual operating conditions of clinical ^90^Sr-^90^Y generators. In practical systems, the activity of ^90^Sr is typically several orders of magnitude higher than that of ^90^Y, thus dominating the system. In contrast, the Sr/Y feed ratios commonly reported in the literature range from 1:1 to 4000:1, which are generally lower than those encountered under generator conditions. In addition, most inorganic ion exchangers were originally developed for the removal of Sr^2+^ from nuclear waste streams, while relatively fewer studies have focused on the separation and recovery of trace ^90^Y from high-concentration ^90^Sr systems. Overall, a methodological gap still exists between conventional experimental setups and practical generator operation, particularly in terms of separation direction and radionuclide ratio.

Finally, in terms of environmental impact and sustainability, the preparation of certain materials involves toxic solvents or expensive rare metal doping, and the final disposal and regeneration of saturated adsorbents may lead to secondary pollution. Moving forward, greater attention should be directed toward the development of natural or renewable matrices, alongside the exploration of green production pathways with low energy consumption and low pollution.

Based on the aforementioned challenges, two research directions appear particularly urgent. First, the transition from empirical screening to rational design, through the integration of multiscale simulations with high-throughput experimental validation, is needed to accelerate the discovery of materials that simultaneously satisfy selectivity, stability, and scalability requirements. Second, future work must move beyond non-radioactive simulations using stable isotopes toward dynamic validation under clinically representative conditions, including prolonged ^90^Sr radiation exposure, multi-cycle operation, and the presence of competing ions at realistic concentrations. Only through such rigorous benchmarking can the most promising candidates identified in this review, namely PAA, MSC, sodium birnessite, and select bio-based platforms, be advanced from laboratory prototypes to reliable clinical ^90^Sr-^90^Y generators.

Solid-phase materials for ^90^Sr-^90^Y generators are undergoing a transition from empirical screening to rational design. Bridging the remaining gap between laboratory performance and clinical reliability will require sustained integration of materials science, radiochemistry, and clinical requirements. As global demand for ^90^Y in nuclear medicine continues to grow, addressing this challenge is both a technical imperative and a strategic priority for the radiopharmaceutical supply chain.

## Figures and Tables

**Table 1:** Comparison of extraction chromatographic systems reported for ^90^Sr-^90^Y separation.

Extractant	Support	Medium/Acid	Q_Y_ (mg·g^−1^)	Q_Sr_ (mg·g^−1^)	SF_Y/Sr_	^90^Sr Breakthrough (^90^Sr/^90^Y)	Recovery of Y (%)	Radiological Validation	Ref.
HDEHP	PTFE particles	HCl	–	–	-	10^−6^	>60	2.5 mCi	[7]
HDEHP (primary) + CMPO	Amberchrom CG-71 + TBP	HNO_3_	–	–	-	10^−8^	64–72	5 Ci	[36]
HDEHP	SiO_2_-P	HCl/HNO_3_	23.1–31.8	-	-	-	68.7–100	-	[37,38,39]
Cyanex 572	XAD-4	HCl	9.3	6.1	1.9	-	98	-	[40]
CMPO	SiO_2_-P	HNO_3_	11–13	-	100	-	100	-	[41]
DtBuCH18C6 +[C_2_mim]NTf_2_	XAD-7	H_2_O + Na_2_EDTA	-	14.9 (2)	>5.0 × 10^4^	-	92 (5)	1.0 MBq	[63]
DtBDCH18C6	XAD-7	HNO_3_	-	41.7	>1000	-	100	-	[75]
TODGA	SiO_2_-P	HNO_3_	13.8	-	-	-	100	-	[76]
TODGA	Chromosorb W	HNO_3_ + EDTA	63.7	-	>1000	-	-	-	[10]
TODGA + 1-Dodecanol	SiO_2_-P-F600	HNO_3_	35.4	-	-	-	96.85	-	[82]
DGAA	Silica-gel	HCl/HNO_3_	16.4	-	-	-	>90	-	[84]
C4DGA	SLM	HNO_3_ + EDTA/DOTA			>10^5^			0.05 mCi	[86]

**Table 2:** Representative organic ion-exchange materials reported for Sr/Y separation and ^90^Sr-^90^Y generator applications.

Resin Type	Functional Group	Medium/Eluent	Q_max_/(mg·g^−1^)	KF_Y/Sr_	Y Recovery (%)	Radiological Demonstration	Ref.
SiAaC	-COOH	NaAc-Hac + HCl	119.4	9900	>99	-	[95]
PHA	-CONHOH	HCl + Acetate buffer	-	-	99.9	-	[96]
Dowex-1	Quaternary Ammonium	(NH_4_)_2_CO_3_	-	-	~100	-	[97]
Chelex-100	Iminodiacetate	NH_4_NO_3_ + HNO_3_	5.5	-	99.4 ± 2.4	-	[98]
Dowex 50 W × 8	-SO_3_H	HNO_3_ + SMP	-	50	>90	7.7 GBq·g^−1 1^	[99]

^1^ Yield of ^90^Y activity per unit mass of irradiated fuel.

**Table 3:** Representative inorganic ion-exchange materials reported for Sr/Y separation and ^90^Sr-^90^Y generator applications.

Material	Medium/Eluent	K_d,Sr_ (mL·g^−1^)	K_d,Y_ (mL·g^−1^)	SF_Y/Sr_ or SF_Sr/Y_	Qmax (mg·g^−1^)	Recovery of Y (%)	Radiological Validation	Ref.
Sb_2_O_5_-SiO_2_	HNO_3_	183.6	<0.1	>7769	51.8 (1 M HNO_3_) 160.6 (pH = 6, HOAc/NaOAc)	99.7	-	[102]
MnS_2_-Sb_2_S_3_	NaHCO_3_	-	2.11 × 10^5^	6.27 × 10^5^	31.64	>99	-	[108]
Sodium Birnessite	Acid	28,654 ± 392	677 ± 27	>2500	99 ± 2	>80	185 MBq	[13]
HMD	HNO_3_	<5	3812 (0.001 M HNO_3_)	>5 × 10^4^	-	>80	-	[113]
Clinoptilolite	NaOH/ Na_2_CO_3_	10^4^–10^5^	-	>1000	2.2 meq·g^−1^	High	0.1 mCi	[141]
Sodium Nonatitanate		10^5^–10^6^			4.7meq·g^−1^	High		
Sodium Titanosilicate		High			-	High		
SOL-POS ^1^	HNO_3_	10^3^	10^5^	>1000	-	62–81	tracer experiment	[142]
SOL-PSO ^2^						70–97		
Na_2_TiSiO_5_	Water+ EDTA	-	-	-	1.960 meq·g^−1^	~100	-	[144]
Ag_2_S	HCl+ HNO_3_	10.5	286	27 ± 0.2	-	99.1 ± 0.5	-	[145]
Zirconium Vanadate	HCl+ HNO_3_	-	high	-	-	99.7 ± 3.1	tracer experiment	[146]

^1^ SOL-POS: Monohydroxy-organophosphorus functionalized silica gel synthesized via sol–gel method; ^2^ SOL-PSO: Dihydroxy-organophosphorus functionalized silica gel synthesized via sol–gel method.

**Table 4:** Summary of separation performance of various materials for 90Sr-90Y generator applications.

Material	Feed Ratio (Sr/Y)	Medium/Eluent Conditions	Q (mg·g^−1^)	SF_Y/Sr_	Reusability (Cycles)	Ref
HDEHP/SiO_2_-P	2000/1	Sr Removal: 0.1 M HCl Y Removal: 6 M HCl	34.05	1.93 × 10^3^	5	[160]
SiaC ^1^	4000/1	Loading: NaAc-Hac (pH = 6) Sr Removal: 0.2 M NaCl (pH = 3) Y Removal: 0.1 M HCl	119.40	9.9 × 10^3^	6	[95]
DGAA-Si	1/1	Sr Removal: 0.01 M HCl/HNO_3_ Y Removal: 1.2 M HCl	16.27	-	-	[84]
PAA	3333/1	Loading: 0.01 M HNO_3_ Sr Removal: 0.5 M H_2_C_2_O_4_ Y Removal: 1.2 M HCl	-	10^6^	8	[158]
MnS_2_-Sb_2_S_3_	4000/1	Loading and washing: H_2_O Sr Removal: 0.2 M NaCl Y Removal: 0.5 M NaHCO_3_	31.64	2.11 × 10^5^	3	[108]
VNP20009 ^2^	300/1	Loading and Y Removal: 5 mM MES buffer (pH = 6) Sr Removal: 0.5 mM Sodium citrate	145.8	1.1 × 10^5^	10	[159]
Aspergillus terreus	1/1	Loading and Sr removing: H_2_O Y Removing: 0.1 M HNO_3_	63.00	1.57 × 10^4^	3	[161]

^1^ a carboxyl-functionalized mesoporous ion-exchange resin; ^2^ a protein-guided bioadsorbent based on Salmonella typhimurium engineered to display the lanthanide-binding protein LanM on its surface.

## Data Availability

No new data were created or analyzed in this study. Data sharing is not applicable to this article.

## Abbreviations

The following abbreviations are used in this manuscript:

Abbreviation Name	Full Name
NCA	no carrier added
EXC	extraction chromatography
DGA	diglycolamide
ICRP	International Commission on Radiological Protection
HLLW	high-level liquid waste
HDEHP	di(2-ethylhexyl) phosphoric acid
HEH[EHP]	2-Ethylhexylphosphonic acid mono-2-ethylhexyl ester
H[DTMPP]	bis(2,4,4-trimethylpentyl) phosphinic acid
CMPO	octyl(phenyl)-N,N-diisobutylcarbamoylmethylphosphine oxide
DtBuCH18C6	di-tert-butylcyclohexano-18-crown-6
DCH18C6	dicyclohexano-18-crown-6
SiO_2_-P	silica/polymer composites
PS-DVB	polystyrene-divinylbenzene copolymers
K_d_	distribution coefficient
SLM	Supported liquid membrane
PTFE	polytetrafluoroethylene
HFMC	hollow fiber membrane contactor
D	distribution ratio
DF	decontamination factor
CMPS-DB18C6	dibenzo-18-crown-6 functionalized chloromethylated polystyrene
TODGA	N,N,N′,N′-tetraoctyl diglycolamide
T2EHDGA	N,N,N′,N′-tetra-2-ethylhexyldiglycolamide
PS	polysulfone
TiBDGA	N,N,N′,N′-tetraisobutyl-diglycolamide
TCHDGA	N,N,N′,N′-tetracyclohexyldiglycolamide
DGAA-Si	diglycolamic acid-grafted silica adsorbent
C4DGA	calix[4]arene
NPHE	o-nitrophenyl hexyl ether
S-Ch-BEN	sulfonated chitosan-bentonite
PHA	polyhydroxamic acid (PHA)
SMP	sodium trimetaphosphate
IDA	iminodiacetic acid
Sb_2_O_5_	antimony pentoxide
SF	separation factors
C-SbA	crystalline antimonic(V) acid
PAA	polyantimonic acid
DFT	density functional theory
PAN	polyacrylonitrile
R	removal rate
HMD	hydrated Manganese Dioxide
LDHs	layered double hydroxides
MSC	MnS_2_-Sb_2_S_3_
SbS-1K	a pH-controlled potassium thioantimonate
AOS	average oxidation state
Z4A	4A zeolite
Ca-def HA	calcium-deficient hydroxyapatite
MXenes	metal carbides/nitrides
PEGDA	poly(ethylene glycol) diacrylate
